# Reactive Hyperemia-Triggered Wrist Pulse Analysis for Early Monitoring of Young Men with High Atherosclerotic Risk

**DOI:** 10.3390/diagnostics11101918

**Published:** 2021-10-16

**Authors:** Jian-Jung Chen, Hsien-Tsai Wu, Bagus Haryadi

**Affiliations:** 1 Taichung Tzuchi Hospital, The Buddhist Tzuchi Medical Foundation, Taichung 42743, Taiwan; 2 School of Post-Baccalaureate Chinese Medicine, Tzu Chi University, Hualien 97002, Taiwan; 3 Department of Electrical Engineering, Dong Hwa University, No. 1, Sec. 2, Da Hsueh Rd., Hualien 97401, Taiwan; hsientsaiwu@gmail.com (H.-T.W.); bagus.haryadi@fisika.uad.ac.id (B.H.); 4 Department of Physics, Universitas Ahmad Dahlan, Jendral A. Yani Street, Kragilan, Tamanan, Kec. Banguntapan, Bantul, Yogyakarta 55191, Indonesia

**Keywords:** wrist pressure pulse (WPP), reactive hyperemia, cardiovascular disease (CVD), endothelial dysfunction (ED), dilation index (DI), heart rate variability (HRV)

## Abstract

The high prevalence of cardiovascular disease in young adults has raised significant concern regarding the early identification of risk factors to allow for timely intervention. This study aimed to identify young males at risk of atherosclerosis using a noninvasive instrument and an initial application percussion entropy analysis of the wrist pressure pulse (WPP). In total, 49 young males aged 18 to 28, without any known history of vascular disease, were recruited. Blood samples were obtained whereby a TC/HDL cutoff value of 4 was used to divide the young men into low-risk (Group 1, TC/HDL < 4, *N* = 32) and high-risk (Group 2, TC/HDL ≥ 4, *N* = 17) groups regarding atherosclerosis. The reactive hyperemia-triggered WPPs were measured using a modified air-pressure-sensing system (MAPSS). The dilation index (DI) of the endothelial function and percussion entropy index (PEI) of the heart rate variability (HRV) assessments, calculated using pragmatic signal-processing techniques, were compared between the two groups. The nonparametric Mann–Whitney *U* test showed that the DI and PEI of the two groups showed statistical differences (both *p* < 0.05). Not only could the MAPSS assess endothelial function and HRV in young males, but the results also showed that waist circumference and PEI may serve as indicators for the early identification of young males at risk of atherosclerosis.

## 1. Introduction

In general, cardiovascular disease should not impact young adults (e.g., ages 18–28). Though the majority of young adults are free from cardiovascular disease (CVD), this group is gradually acquiring more CVD risk factors, especially due to unhealthy lifestyle factors, such as low exercise, lack of sleep, and poor dietary habits [1]. When attempting to optimize public health, two essential points require attention: expanding preventive efforts beyond high-risk groups; and encouraging young adults to take up healthier lifestyles [2]. Recent studies have further demonstrated that most noncommunicable diseases are related to an unhealthy lifestyle: (1) cigarette smoking; (2) hypertension; (3) hyperglycemia; (4) dyslipidemia; (5) obesity; (6) physical inactivity; and (7) poor nutrition [3]. In [4], options for starting strong in life were proposed, and health services were redesigned in line with these. It is known that metabolic syndrome increases not only the chance of developing type 2 diabetes mellitus, but also that of developing CVD [5]. Obesity and smoking have also been reported as the main risk factors for blood vessel plaque in young men [6]. However, there is insufficient evidence of efficacy in the longer-term for improving smoking rates, nutrition behaviors, alcohol intake, physical activity levels, and/or obesity in young adults [7]. The total serum cholesterol/high-density lipoprotein (TC/HDL) ratio is regarded as an atherosclerotic risk factor of CVD, and is related to insulin resistance syndrome. This is the reason the TC/HDL ratio is associated with more substantial alterations in metabolic indices predictive of ischemic heart disease risk than variations in the low-density lipoprotein/high-density lipoprotein (LDL/HDL) ratio [8]. That study also reported that elderly subjects with low HDL cholesterol had a higher risk of coronary heart disease than subjects with optimal lipid profiles, but no difference in survival after a median 10 years of follow-up [9]. More importantly, [10] found that subjects with a TC/HDL ratio ≥ 4 had an odds ratio of 1.74 compared to those with a TC/HDL ratio < 4, for subjects who were relatively elderly, with a mean age of 48.4  ±  11.5 years. In addition, some studies have also found that the TC/HDL ratio is a good predictor of cardiovascular disease and nonalcoholic fatty liver, which is closely related to CVD [11,12]. It is well known that a good lifestyle (e.g., not smoking, eating healthy, exercising frequently, and sleeping well, which are reflected in blood sampling data) can keep most people healthier for a longer time. However, despite significant progress in CVD prevention, the rate of myocardial infarctions has still not declined in young adults [12]. It is not a simple matter for young adults to take care of the health of their bodies, and it also depends on them understanding how to assess their health (e.g., in terms of their cardiovascular system) and knowing what steps are needed to improve their health and substantially delay CVD. It is urgent that young adults understand how to achieve and maintain vascular health for CVD prevention (e.g., education, and encouraging young adults to adopt healthier lifestyles) [12].

A recent study [13] addressed the effect of body composition on the cardiovascular system, especially focusing on vascular endothelial function and heart rate variability (HRV). Furthermore, a previous study regarded vascular endothelial dysfunction (ED) as an early predictor of the development of atherosclerosis and cardiovascular diseases [14]. Moreover, ED was found to be associated with cardiovascular and metabolic diseases in [15]. The noninvasive flow-mediated dilation test of ultrasonic assessment that was reported in [16] was incorporated into a forearm occlusion and subsequent reactive hyperemia (RH), which promotes nitric oxide production and vasodilation of the brachial artery. ED was associated with CVD events in a high-end hospital, considering 12 young men who underwent an ED assessment [17]. In addition, the review in [18] described up-to-date information on normal vascular ED, and traditional and novel potential biomarkers of ED. The traditional evaluation index for HRV in the frequency domain (i.e., the low frequency power (LFP)/high frequency power (HFP) ratio, (LHR)) [19] was examined for comparison. Recently, the authors of [20] proposed a new percussion entropy index (PEI) to assess diabetic autonomic nervous dysfunction. As a small-scale multiscale entropy index, the PEI also reflects the baroreflex sensitivity (BRS) function, based on cross entropy-based methods for comparing HRV using RR interval (RRI) datasets [21]. Furthermore, in [22], the peak-to-peak interval (PPI) was considered an alternate way to assess HRV using PEI (i.e., PEI_DVP_) without electrocardiography for the prediction of diabetic peripheral neuropathy. However, there is still no reliable PEI application of the wrist pressure pulse (WPP) for early identification of young men with high atherosclerotic risk. Therefore, a modified air pressure sensing system (MAPSS) with DI computation (e.g., without EEMD calculation) [23] and a new HRV index (e.g., with PEI computation) were developed in response to the urgent need for early identification of young men with high atherosclerotic risk.

The aim of this study was two-fold. First, a feasible endothelial function and HRV assessment system that uses a wrist waveform with a reactive hyperemia-triggered approach was established. Second, the current study attempted to validate the application of the system for early identification of young men at high atherosclerotic risk of CVD.

This manuscript is organized as follows. Section 2 presents the WPP acquisition system and a new HRV index. The differentiating capability of PEI and DI obtained from the WPP signals are discussed in Section 3. Subsequently, risk factors of young men with high atherosclerotic risk of CVD are outlined. The findings are discussed in Section 4. Section 5 concludes the manuscript.

## 2. Physiological Signal Acquisition and Medical Indexes

### 2.1. Study Population and Grouping

Between May 2011 and May 2012, a total of 49 young male participants aged between 18 and 28 were recruited. Individuals with diabetes, cardiovascular diseases, or a history of related diseases, those who had received medical treatment within the last three months, and those who engaged in cigarette smoking were excluded from the study. All participants were required to fast for 8 h. On the day of examination, the participants were taken to the outpatient clinic department in Hualien hospital for medical assessment and blood samples, including glycosylated hemoglobin (HbA1c), fasting blood sugar concentration, high-density lipoprotein (HDL), low-density lipoprotein (LDL), triglycerides (TGs), and total cholesterol. Physiological data (such as age, body weight, height, and waist circumference) of the participants were also recorded. This study was conducted in accordance with the Helsinki Declaration, and the Human Research Committee of Hualien Hospital (Hualien City, Taiwan) [23].

Hormonal changes associated with menstruation in females may contribute to the stiffness of blood vessels. In addition, age and diabetes are also risk factors for atherosclerosis. This is the reason only young and apparently healthy men were enrolled in the study. To further delineate the young male subjects in our study, we adopted this ratio, and also chose a cutoff value of 4 to divide our tested young men into the low-risk (Group 1, TC/HDL < 4) and high-risk (Group 2, TC/HDL ≥ 4) groups for atherosclerosis. Subsequently, to better verify the capabilities of the method for monitoring and/or tracking atherosclerosis, an older age group (Group 3: 30 middle-aged men) was added for comparison.

### 2.2. Signals Acquisition System Description

**Figure 1:**
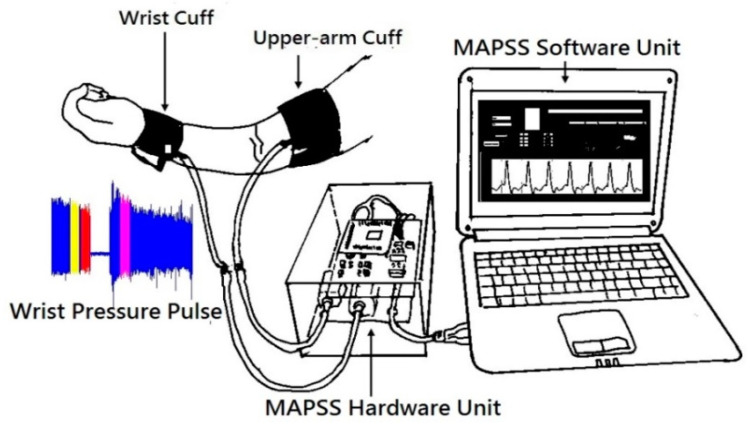
Schematic illustration of the modified air pressure sensing system (MAPSS). The MAPSS hardware unit consists of an air pressure sensing unit, wrist/upper-arm air pump motor, and mixed signal processing unit. The analyzing software executes every six seconds after receiving the data from the mixed signal processing unit, which has a sampling rate of 500 Hz in the MAPSS software unit.

When the medical assessment was finished, the subject was taken to a health clinic for signal acquisition. All subjects provided written informed consent before measurements were taken. Subsequently, resting blood pressure was measured with the left arm in a supine position using an oscillometric device (BP3AG1, Microlife, Taipei, Taiwan). In this study, we adopted a MAPSS for endothelial function and HRV assessment. A MAPSS is composed of two sets of pressure cuffs (i.e., a wrist cuff and an upper-arm cuff), a hardware control unit, and medical indexes computation software (Figure 1).

**Figure 2:**
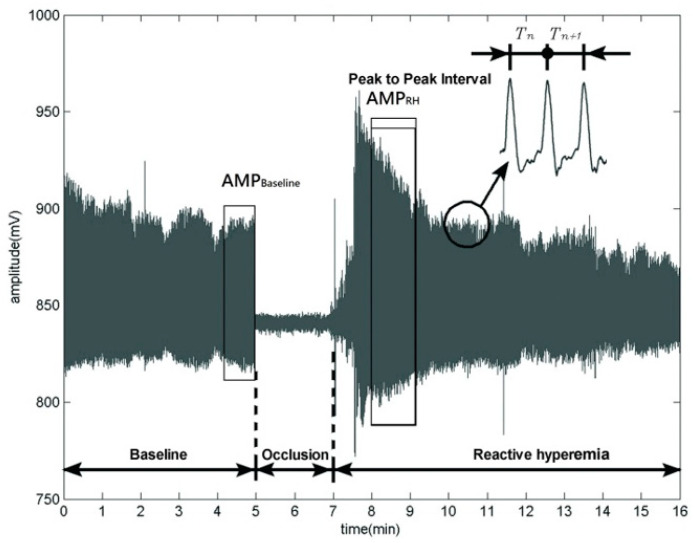
Wrist waveform extraction in the first 5 min (e.g., baseline). During the occlusion phase, the pressure cuff on the upper left arm was inflated to 200 mmHg to stop the blood flow for two minutes. For those two minutes, the wrist waveform was very small. When the arm pressure cuff deflated to 0 mmHg, the wrist waveform had a larger amplitude than baseline, and the peak-to-peak interval (PPI) time series changed under the effects of reactive hyperemia. The overall signal acquisition time was 16 min.

During the wrist waveform measurement, each participant was asked to lie flat and remain in a relaxed posture for 4 min in a quiet space at 25 ± 1 °C, and the waveform was acquired using the MAPSS. In brief, the wrist cuff pressure was maintained at 40 mmHg for wrist waveform extraction on the left wrist for five minutes as a baseline. Subsequently, the upper-arm cuff was inflated to 200 mmHg to stop the blood flow for two minutes. The wrist waveform extraction on the left wrist was small, during an occlusive period. Subsequently, the deflation valve in the hardware control unit deflated on the upper-arm cuff. The wrist pressure pulse extraction was performed for nine minutes with the wrist cuff, during post-occlusive reactive hyperemia (RH). The amplitude and frequency information of the waveforms showed obvious changes (Figure 2).

In this study, the upper-arm cuff was mainly used to trigger reactive hyperemia, and the wrist cuff was used to capture the pulse signals. The detailed structure and principles of the MAPSS can be found in [23,24].

### 2.3. Medical Indexes from Physiological Signals

#### 2.3.1. Amplitude Variation for Vascular Relaxation Assessment

- Endothelial Function Assessment

The endothelial function assessment for vascular relaxation was represented by the quantification of reactive hyperemia (RH), which is a vasodilatory phenomenon brought about by the enhanced release of endothelium-derived relaxing factor (i.e., nitric oxide (NO)) [25] from endothelium cells through an increase in shear stress during reperfusion after a short period of ischemia (e.g., the upper-arm cuff was inflated to 200 mmHg to stop the blood flow in our study).

- Medical Index for Endothelial Function

Accordingly, the mean amplitudes of the signals within representative 1 min periods (about 60–120 heart beats) during the fifth and ninth minutes after the beginning of wrist waveform collection (via air pressure sensing) were chosen from the baseline and RH phase (Figure 1), respectively, and labeled as AMP_Baseline_ and AMP_RH_. The 60-s RH phase was divided into 6-s intervals, from which the highest peak value was chosen as the mean peak value during the RH phase, i.e., AMP_RH_. The DI ratio = the mean peak value during the RH phase/the mean peak value during the baseline phase. The dilation index (DI) of the endothelial function assessment was defined as [23].

(1) $$\mathrm{DI}=\frac{{\mathrm{AMP}}_{\mathrm{RH}}}{{\mathrm{AMP}}_{\mathrm{Baseline}}}.$$

The assessment of vascular function was based on reactive hyperemia (RH) that produced different arterial pulse wave amplitudes for comparing with the mean baseline amplitude (i.e., the DI). The arterial waveform was not used for analysis in this study.

#### 2.3.2. Periods Variation for HRV Assessment

Heart rate variability (HRV) analysis is a critical method [26] for evaluating autonomic nervous functions. Two well-established HRV analysis methods were adopted in this study (frequency domain analysis and the entropy method).

- LFP/HFP ratio (LHR)

In frequency domain analysis, the R–R interval (RRI) is recorded using an electrocardiogram, and the energy distribution of the RRI under various frequencies is acquired by using the Fourier transform. Sun et al. verified that the RRI series is consistent with the peak-to-peak interval (PPI) series of the digital volume pulse [27]. Therefore, we measured the wrist pressure pulse 9 min after reactive hyperemia using the APSS, and the fast Fourier transform (FFT) analysis was utilized on the PPI series from the RH phase. The selection of the frequency band for nervous activity was from [24]. As the common indicator of HRV with frequency domain analysis, the LHR reflects the equilibrium of the sympathetic and parasympathetic nerves [24].

- Percussion entropy index

A previous study [22] reported on the application of the speedy percussion entropy index (i.e., PEI_DVP_), which adopted the digital volume pulse after ensemble empirical mode decomposition in assessing the complexity of baroreflex sensitivity for age-controlled healthy and diabetic subjects. Accordingly, the baroreflex sensitivity delay between the amplitude series and PPI series from the wrist pressure pulse (WPP) in the RH phase in the current study could be set as 1, delayed for healthy young men, and the new speedy percussion entropy index (i.e., PEI_WPP_) was then calculated as follows:

(2) $${\mathrm{PEI}}_{\mathrm{WPP}}\left(m,S1\right)=ln\left[\frac{\sum_{s=1}^{\mathrm{Si}}P_{s}^{m}}{\sum_{s=1}^{\mathrm{Si}}P_{s}^{m+1}}\right],ln:\mathrm{natural}\;\mathrm{logarithmic}\;\mathrm{operation},$$

(3) $${\mathrm{where}\;P}_{s}^{m}=\frac{1}{\left(n-m-s+1\right)}\sum_{i=1}^{n-m-s+1}\mathrm{count}\left(i\right),$$

(4) $${\mathrm{and}\;P}_{s}^{m+1}=\frac{1}{\left(n-m-s+2\right)}\sum_{i=1}^{n-m-s+2}\mathrm{count}\left(i\right),$$

where n = 700 (e.g., for 9 min WPP in RH phase), m = 2, and s = 1 for healthy young men in the current study. $P_{s=1}^{2}$ and $P_{s=1}^{3}$ were the percussion rates with different lengths of fluctuation vector, 2 and 3, respectively. The percussion count increased when the two compared patterns of fluctuation were identical. The high percussion rate represented high similarity in the pattern of fluctuation, as described in [20].

### 2.4. Signal Analysis and Statistical Analysis

The DI and PEI computation were conducted using the academic software LabVIEW by National Instruments (Austin, TX, USA) in MAPSS. All data are expressed as the mean ± standard deviation (SD). SPSS statistical software (SPSS Inc., Chicago, IL, USA) was used for nonparametric statistical tests. The statistical analysis was performed with a confidence level of 95%. Moreover, the *p* value had to be smaller than 0.05 to achieve statistical significance. The young men’s characteristics, blood sample tests, and calculated indices were compared between those with or without TC/HDL ≥ 4, and subjected to statistical analysis using the nonparametric Mann-Whitney *U* test [28]. The effects of risk factors for atherosclerosis on PEI and DI were evaluated by multivariate logistic regression analyses, with the probability of developing a high-risk event (i.e., TC/HDL ≥ 4) for the tested young men.

## 3. Results

### 3.1. Characteristics of the Study Subjects

Among 49 young men (Group 1 and Group 2), 11 subjects had a waist circumference larger than 90 cm, 7 had BMI > 27, 19 had hypercholesterolemia (e.g., LDL > 120), and 17 subjects had TC/HDL > 4. The demographic, anthropometric, hemodynamic, and serum biochemical parameters of all the study participants are shown in Table 1. The average age of the participants was under 23 (22.36 ± 3.01), and other blood parameter values were within normal ranges. In terms of mean values, waist circumference and body mass index measurements were just lower than the upper acceptable limits. On the other hand, TC/HDL (4.02 ± 0.99) was not within the normal range and had wide variations.

### 3.2. Grouping of the Young Men by TC/HDL and Medical Indexes

A TC/HDL ratio of 4 is recognized as a criterion for atherosclerosis risk [10]. Therefore, the young male subjects were divided into two groups in the current study, with middle-aged men for comparison. The participants who had TC/HDL ratios less than 4 were in Group 1 (*N* = 32) and Group 3 (*N* = 30), whereas those who did not meet that criterion served as Group 2 (*N* = 17) (Table 2). A remarkable difference between the two groups (Group 1 versus Group 2) in terms of the TC/HDL ratio was noted (*p* < 0.001). Furthermore, significant differences in high-density lipoprotein cholesterol, low-density lipoprotein cholesterol, total serum cholesterol, and triglycerides were also found between the two groups (Group 1 vs. Group 2, all *p* < 0.017) (Table 2). The LHR values of the participants in Group 1 were higher than those of the participants in Group 2 (0.92 ± 0.47 vs. 0.86 ± 0.62, *p* = 0.377), however, the differences were not statistically significant among the three groups. More importantly, young men with a high atherosclerotic risk of CVD had significantly lower PEI_WPP_ and lower DIs than those without a high atherosclerotic risk of CVD, as determined by the MAPSS, with 56.06 ± 11.17% vs. 75.56 ± 8.96%, *p* = 0.001, and 1.45 ± 0.27 vs. 1.75 ± 0.39, *p* = 0.006, respectively (Table 2). Unexpectedly, Group 2 had significantly lower PEI_WPP_ than Group 3 (Table 2).

### 3.3. Effects of Risk Factors

From Table 3, the fitted logistic regression model, adjusted for age, body mass index, low-density lipoprotein cholesterol, and triglycerides, is shown:

logit (P) = −1.548 + 0.138 × Waist Circumference − 0.163 × PEI_WPP_ (5)

The values of logit (P) = ln (P/(1 − P)), where ln is defined as the natural logarithm operation, and P is the probability of developing a high atherosclerotic risk of a CVD event (i.e., TC/HDL ≥ 4) for the tested young men. Seventeen of the 49 enrolled young men had a high atherosclerotic risk of a CVD event in the current study. The odds ratio for PEI_WPP_ in (2) was 0.849. This indicates that for the given values of PEI_WPP_, the odds of developing high atherosclerotic risk of CVD in the tested young men was multiplied by 0.849 for every 1% increase in PEI_WPP_ (Table 3).

## 4. Discussion

Cardiovascular disease (CVD) has become the most important contributor to deaths from noncommunicable diseases worldwide. There is an urgent need to better prevent the disease among young adults [1,2]. A recent study addressed research challenges and opportunities related to the cardiovascular health of young adults (i.e., 18–39 years old). The study collected real data documenting a lack of progress in CVD prevention in the young male group, as evidenced by the significant prevalence of risk factors [29]. According to the results in [30], arteries in adults can be progressively damaged until the heart’s arteries are also damaged and, hence, new clinical syndromes gradually emerge after the age of 30. On the other hand, the health-enhancing impact of physical activity on cardiovascular function has been highlighted in healthy young adults aged 18–25 [31]. The findings in [32] also provided evidence for enhanced bioavailable curcumin improving homocysteine and high-density lipoprotein concentrations, which may promote favorable cardiovascular health in young (18–35) obese men (body mass index ≥ 30.0 kg/m^2^), in which endothelial function was assessed with the reactive hyperemia index. Furthermore, the endothelial function assessment for vascular relaxation is represented by the quantification of reactive hyperemia (RH) [24], and was validated for type 2 diabetics in [23]. As reported in [13], a reasonable inference is that the metabolic risk factors associated with cardiovascular problems could reveal subtle changes in vascular and autonomic function as a result of arteriosclerosis in unhealthy young men. Moreover, by using an MAPSS, young men may come to better understand vascular endothelial and HRV dysfunction of the body toward CVD prevention (Figure 1 and Table 2). In addition, another study [33] demonstrated that arterial wave reflections in young adults with obesity could have shorter waveform periods than those of elders. That is, it is reasonable to assume that nine minutes of reactive hyperemia-triggered [34] wrist waveform in MAPSS could reflect the HRV capability of young men with or without high atherosclerotic risk of CVD. As shown in Table 3, waist circumference (as a vital sign in clinical practice) was the significant risk factor, with an odds ratio of 1.148 for incident risk analysis of high atherosclerotic risk of CVD for young men.

In general, CVD should not be a significant problem for young adults. Consistent with the results in [2], recent research has further demonstrated that metabolic syndrome is a cluster of clinical disorders whose development is promoted by various factors, including having unhealthy lifestyle habits with a large waist circumference, dyslipidemia, glucose intolerance, and high hypertension. It is known that metabolic syndrome not only increases the chances of developing type 2 diabetes mellitus, but also CVD [1,35]. As reported, cardiovascular function varies with age because of physiological and pathological factors: the results in one study [36] examined the relationships of longitudinal changes in cardiovascular function with changes in metabolic traits for 5779 participants (mean age, 49.8 ± 14.5 years; 54% female). Moreover, changes in eating and metabolism, autonomic function, and sleep function have been checked across several neurodegenerative conditions [37]. In addition, the results in [38] confirmed that efforts to prevent a decline in cardiovascular health are difficult, and CVD outcomes should reflect established clinical methods of cardiometabolic risk reduction in patients: smoking cessation, weight management, and adequate blood pressure and lipid control. Cardiovascular function in young men was found to be different in those with unhealthy lifestyle habits, namely those with a large waist circumference, dyslipidemia, glucose intolerance, and high hypertension. We did not find statistically significant differences based on waist circumference, fasting plasma glucose, glycated hemoglobin, systolic blood pressure, or diastolic blood pressure between healthy young men (Group 1) and young men at high atherosclerotic risk of atherosclerosis (Group 2) (Table 2). Dyslipidemia was the exception (high-density lipoprotein cholesterol, low-density lipoprotein cholesterol, total serum cholesterol, and triglycerides). TC/HDL ≥ 4 is not only a good predictor of CVD [10], but is also consistent with the effects found to be associated with dyslipidemia in young men in the current study. Young men (e.g., age 18–28) at high atherosclerotic risk (with TC/HDL ≥ 4) had lower dilation indices and lower PEI_WPP_ values (Table 2), which reflect dysfunction of vascular relaxation (i.e., endothelial function) and HRV, respectively (Table 1 and Table 2). According to (1), the wrist waveform has a larger amplitude than the baseline [16], and according to (2), the peak-to-peak interval (PPI) time series changes [18] under the effects of reactive hyperemia for young men under the age of 30. This study first investigated PEI_WPP_ (e.g., a protective factor) for effects in HRV measures in young men (Table 3). A recent review study [34] emphasized that RH has the potential to provide research and clinical practice guidelines for investigating microvascular function and waveform regulation between the lifespan and health span. The findings in the current study could serve as a proof-of-concept implementation of the review study [34].

The story of Bian Que and King Wei Wen in ancient China states that, “Treating diseases is not better than preventing them”. One day, King Wei Wen asked Bian Que, a famous doctor, “The three brothers in your family are all good at medicine. Which one is the best?” Bian Que replied: “The eldest brother is the best and I am the worst.” The reason for this is that his eldest brother treated the illness before its onset, although most people thought that he could only cure minor diseases. The phrase “prevention is better than a cure” is often attributed to the Dutch philosopher Desiderius Erasmus, at around 1500, from the historical perspective. It is interesting that Hippocrates and Aclepius, in ancient Greece, had the same idea. Promotion of healthy lifestyles and the prevention of ill health are fundamental to public health. Therefore, MAPSS can also contribute to early identification of young males at risk of atherosclerosis with PEI, TC/HDL, and waist circumference evidence, so that these young men can understand their cardiovascular system, and be encouraged to live healthier lifestyles [3,39,40].

## 5. Conclusions, Limitations, and Future Research

This study presented initial results related to the development of a modified system that can be used to stratify atherosclerotic risk in healthy and young subjects. The wrist waveform during post-occlusive reactive hyperemia was evaluated for simultaneous assessments (DI and PEI) of atherosclerosis, which individually used the amplitude and HRV information of waveforms. The findings suggest that the waist circumference and PEI_WPP_ are both important risk and protective factors for the early identification of young men at high atherosclerotic risk of CVD and, thus, a large waist circumference and a low PEI_WPP_ indicate an increased risk of future atherosclerosis.

Several limitations are relevant for this study. First, this study was not a wide-ranging clinical study, and therefore, the number of participants was limited, thus affecting the statistical rigor. Second, the wrist waveform measured during post-occlusive reactive hyperemia using MAPSS was probably affected by vibrations, conversation, and emotional stress. Therefore, we described the experimental procedure to the participants to ease their tension, and asked them to quietly rest for 5 min before the test began, thereby reducing measurement errors caused by emotional factors and external interference. Thirdly, this study did not actually verify via pharmacology [41] that endothelial function, strictly per the nitric oxide signaling pathway, occurs during hyperemia using our particular study approach. Finally, young men with CVD were not included in the study. The application of this system in young men with CVD is another important field to be explored.

Physiological signals can be used in various clinical disciplines, but novel signal-processing techniques and noninvasive instruments are priorities for preventive medicine. More importantly, the results of this study could be relevant globally, especially when addressing COVID-19 and lifestyle issues, and the overall habits of the younger population.

## Figures and Tables

**Table 1:** Demographic, anthropometric, hemodynamic, and serum biochemical parameters of the study subjects (*N* = 49).

Parameter	Value			Normal Value
Age (year)	22.36	±	3.01	-
Body Height (cm)	172.51	±	5.87	-
Body Weight (kg)	70.45	±	14.42	-
Waist Circumference (cm)	84.87	±	12.05	<90, for man
Body mass index (kg/cm^2^)	23.95	±	4.05	18.5 ≤ BMI < 24
Systolic Blood Pressure (mmHg)	119.75	±	12.38	<135
Diastolic Blood Pressure (mmHg)	71.99	±	7.87	<85
Pulse Pressure (mmHg)	47.48	±	9.01	20~60
High-density lipoprotein (mg/dL)	44.68	±	7.65	>40, for man
Low-density lipoprotein (mg/dL)	107.74	±	31.94	<110
Total serum cholesterol (mg/dL)	175.33	±	34.09	<200
Triglyceride (mg/dL)	93.28	±	63.86	<150
TC/HDL Ratio	4.02	±	0.99	≤4
Fasting Blood Sugar (mg/dL)	94.05	±	6.79	<120
HbA1c (%)	5.45	±	0.31	<6.5

Values expressed as mean ± standard deviation. TC/HDL Ratio = Total serum cholesterol/High-density lipoprotein; HbA1c = Glycated Hemoglobin.

**Table 2:** Comparison of the parameters for healthy young men (Group 1), young men at high atherosclerotic risk of CVD (Group 2), and middle-aged men (Group 3) in the current study.

Parameters	Group 1	Group 2	Group 3
Age (year)	22.96 ± 2.46	22.72 ± 3.30	52.04 ± 9.98 ^††^
Body Height (cm)	172.08 ± 5.57	170.89 ± 5.32	161.91 ± 8.19 ^†^
Body Weight (kg)	67.58 ± 9.62	74.67 ± 16.82	65.79 ± 15.82
WC (cm)	83.34 ± 8.53	89.32 ± 13.91	87.58 ± 10.38
BMI (kg/cm^2^)	22.76 ± 2.68	25.51 ± 5.27	24.81 ± 3.49
SBP (mmHg)	119.00 ± 13.14	119.83 ± 11.74	127.43 ± 16.59
DBP (mmHg)	72.16 ± 7.48	71.50 ± 8.21	74.59 ± 12.52
HDL (mg/dL)	47.52 ± 7.51	42.83 ± 6.91	51.97 ± 19.52
LDL (mg/dL)	86.68 ± 17.56	134.92 ± 25.46 **	112.98 ± 27.89 ^†^
TC (mg/dL)	154.44 ± 19.69	201.84 ± 30.86 **	188.88 ± 42.69
TG (mg/dL)	91.48 ± 26.79	121.17 ± 85.98	110.83 ± 96.79
FPG (mg/dL)	92.48 ± 4.95	94.56 ± 8.38	105.34 ± 21.65
HbA1c (%)	5.47 ± 0.28	5.51 ± 0.30	6.02 ± 0.58 ^†^
TC/HDL Ratio	3.48 ± 0.40	5.09 ± 0.86 **	3.98 ± 1.70
DI	1.75 ± 0.39	1.45 ± 0.27 *	1.31 ± 0.48
LHR	0.92 ± 0.47	0.86 ± 0.62	0.89 ± 0.87
PEI_WPP_ (%)	70.56 ± 8.96	56.06 ± 11.17 *	68.37 ± 9.17 ^†^

Group 1: young men with TC/HDL < 4 (*N* = 32), Group 2: young men with TC/HDL ≥ 4 (*N* = 17), and Group 3: middle-aged men (*N* = 30). WC: waist circumference measurement; BMI: body mass index, a simple calculator using a person’s height and weight; SBP: systolic blood pressure; DBP: diastolic blood pressure; HDL: high-density lipoprotein cholesterol; LDL: low-density lipoprotein cholesterol; TC: total serum cholesterol; TG: triglycerides; FPG: fasting plasma glucose HbA1c: glycated hemoglobin; DI: dilation index [23]; LHR: LFP/HFP ratio; PEI_WPP_: percussion entropy index for wrist pressure pulse. * *p* < 0.017 and ** *p* < 0.001, were regarded as statistically significant between the Group 1 vs. Group 2, using nonparametric Mann–Whitney U test. ^†^ *p* < 0.017 Group 2 vs. Group 3; ^††^ *p* < 0.001 Group 2 vs. Group 3.

**Table 3:** Incident risk analysis of high atherosclerotic risk of CVD with two factors.

Risk Factors	Coefficient	*p*	OR	95% CI for OR
WC	0.138	0.015	1.148	1.027–1.283
PEI_WPP_	−0.163	0.003	0.849	0.761–0.948
Constant	−1.548	0.076	0.213	-

OR: odds ratio; CI: confidence interval; WC: Waist Circumference; PEI_WPP_: percussion entropy index in (2). The logistic regression analysis was adopted in SPSS. A *p*-value < 0.05 was considered statistically significant for the test factor. R2 for logistic regression: Cox–Snell R2 = 0.434 and Nagelkerke R2 = 0.599. Overall percentage in classification table = 91.8% for the fitted model in (5).

## Data Availability

Not applicable.

## Abbreviations

BRS: baroreflex sensitivity; COVID-19: coronavirus disease 2019; CVD: cardiovascular disease; DI: dilation index; ED: endothelial dysfunction; EEMD: ensemble empirical mode decomposition; HDL: high-density lipoprotein; HRV: heart rate variability; LHR: the low frequency power/high frequency power ratio; MAPSS: modified air pressure sensing system; PEI: percussion entropy index; RH: reactive hyperemia; TC: total serum cholesterol; WC: waist circumference; WPP: wrist pressure pulse.
